# A method for *in vivo* evaluation of α-glucosidase inhibition using *Drosophila*

**DOI:** 10.1016/j.mex.2023.102373

**Published:** 2023-09-09

**Authors:** Nattapong Wongchum, Alongklod Tanomtong, Somchai Pinlaor, Chatmongkon Suwannapoom, Ananya Dechakhamphu

**Affiliations:** a Biology Program, Faculty of Science, Ubon Ratchathani Rajabhat University, Ubonratchathani 34000 Thailand; b Biology Program, Faculty of Science, Khon Kaen University, Khon Kaen 40002, Thailand; c Department of Parasitology, Faculty of Medicine, Khon Kaen University, Khon Kaen 40002, Thailand; d Department of Fishery, School of Agriculture and Natural Resources, University of Phayao, Phayao 56000, Thailand; e Aesthetic Sciences and Health Program, Faculty of Thai Traditional and Alternative Medicine, Ubon Ratchathani Rajabhat University, Ubonratchathani 34000 Thailand; f Thai Traditional Medicine Program, Faculty of Thai Traditional and Alternative Medicine, Ubon Ratchathani Rajabhat University, Ubonratchathani 34000 Thailand

**Keywords:** Animal model, Inhibition assay, In vivo screening, Anti-glucosidase activity, Anti-diabetic assay, Evaluating the inhibition of active compound on α-glucosidase activity using *Drosophila* as a model

## Abstract

The development of α-glucosidase inhibitors is essential for the prevention of type II diabetes. Previous research has investigated *in vitro* inhibition using isolated α-glucosidase, which may not accurately reflect physical processes. The method presented in this study aims to establish a rapid and inexpensive in vivo method to study the inhibition of α-glucosidase activity using *Drosophila* as a model organism. This method can be used to calculate the IC_50_ value of compounds of interest for inhibition of α-glucosidase activity. The method established in this study can be used for *in vivo* screening of anti-diabetic compounds.

• A rapid and inexpensive *in vivo* method to study the inhibition of α-glucosidase activity.
• This method can be used to calculate the IC_50_ value of compounds of interest for inhibition of α-glucosidase activity.
• This is a useful method for in vivo screening of anti-diabetic compounds.

## Graphical abstract

**Figure:**
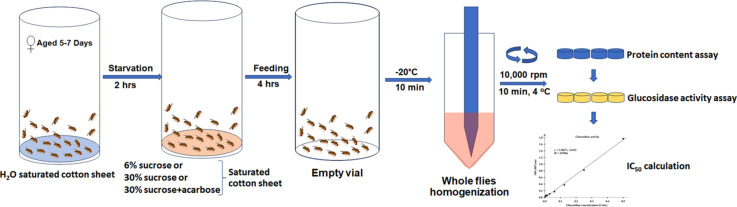

Specifications table

**Table:** 

Subject area:	Pharmacology, Toxicology and Pharmaceutical Science
More specific subject area:	Enzyme activity and inhibition assay.
Name of your method:	Evaluating the inhibition of active compound on α-glucosidase activity using *Drosophila* as a model.
Name and reference of original method:	1. Oboh, G., Ogunsuyi, O. B., Adegbola, D. O., Ademiluyi, A. O., and Oladun, F. L. (2019). Influence of gallic and tannic acid on therapeutic properties of acarbose in vitro and in vivo in *Drosophila melanogaster*. Biomed. J. 42, 317–327. doi:10.1016/j.bj.2019.01.005. [1] 2. Wang, M., Mao, H., Chen, J., Qi, L., & Wang, J. (2022). Ameliorative effect of bayberry leaves proanthocyanidins on high sugar diet induced *Drosophila melanogaster*. Frontiers in Pharmacology, *13*, 1,008,580. doi:10.3389/fphar.2022.1008580. [2]
Resource availability:	α-Glucosidase; Acarbose; Sucrose; 50 mM Potassium Phosphate Buffer (pH 7.0); *p*-nitrophenyl-α-d-glucopyranoside; Bradford reagent; Bovine Serum Albumin (BSA); 96-well plate; Microcentrifuge; Spectrophotometer

## Introduction

Inhibition of α-glucosidase is critical for the management and treatment of type 2 diabetes, a disease characterized by elevated blood glucose, insulin resistance, and relative insulin deficiency [3]. By reducing the rate at which carbohydrates are broken down into simple sugars, α-glucosidase inhibitors reduce postprandial glucose spikes and help control of blood glucose levels, which is an effective way to treat diabetes [4]. In addition to their role in treating diabetes, α-glucosidase inhibitors have also been studied for their potential benefit in the treating other diseases. Some research suggests that slowing carbohydrate digestion may increase feelings of satiety and reduce overall food intake, which in turn would contribute to weight loss [5]. Currently, most studies investigating anti-glucosidase activity are conducted *in vitro*, conditions that do not fully reflect the complexity of *in vivo* conditions. Animal studies are needed to identify and validate new, effective pharmacological agents for the treatment and prevention of diabetes.

*Drosophila melanogaster* is widely recognized as a model organism for the study of metabolic diseases. *Drosophila* has been used to develop diabetes models that closely resemble the features of type 2 diabetes. A model for the induction of T2D in *Drosophila* was developed using a high-sugar diet [6]. As in humans, *Drosophila* contains insulin producing cells (IPCs), insulin-like peptides (dILPs), and an insulin receptor (InR) [7], [8]. Insulin-resistant traits such as metabolic abnormalities, elevated Dilp mRNA levels, and impaired insulin signaling activity can be effectively reproduced in both larval and adult stages of *Drosophila* [9]. Hyperglycemia, insulin resistance, increased fat storage, and shorter lifespan have been studied in *Drosophila* fed a high-sugar diet [10]. In addition, the effects of plant extracts on preventing metabolic disorders associated with excessive sugar consumption in *Drosophila* have been studied [11], [12], [13]. It has been observed that fruit flies contain the enzyme glucosidase for sugar uptake [14]. Therefore, *Drosophila* serves as a useful model organism for studying the inhibition of α-glucosidase activity by compounds of interest. In this study, we developed a protocol for measuring α-glucosidase activity using acarbose as a reference compound. This study's methodology offers several advantages over existing approaches. In particular, testing requires only six hours as opposed to the typical seven to ten days, and the IC_50_ concentration can be determined, a feature not described in any previously published reports. These benefits make it particularly useful for screening compounds of interest *in vivo*. In addition, the established protocol provides a rapid and cost-effective *in vivo* evaluation method for anti-diabetic compounds.

## Method details

Preparation of 50 mM potassium phosphate buffer (pH 7.0 at 25 °C):

1. Add 6.15 mL of 1.0 M potassium phosphate dibasic solution to a beaker.
2. To the same beaker, add 3.85 mL of 1.0 M potassium phosphate monobasic solution.
3. Bring the final volume to 200 mL with ultrapure water.
4. Adjust the pH to 7.0 at 25 °C with 1 M KOH or HCl.

Preparation of the diet solution:

1. To prepare a high-sugar diet (30% sucrose solution), add 30 g sucrose to a volumetric flask and make up the volume to 100 mL with sterile water.
2. To prepare a diet containing 100 M acarbose (MW = 645.604 g/mol), add 0.0065 g acarbose to a volumetric flask and then make up the volume to 100 mL with a 30% sucrose solution. For the preparation of diets containing acarbose at concentrations of 5, 2.5, 1.25, 0.625, 0.3125, 0.156, and 0.078 M, the dilution was calculated using the equation C_1_V_1_ = C_2_V_2_.

### *Drosophila* strain and culture conditions

The wild-type *D. melanogaster* strain Oregon-R-C was used in this study. Flies were maintained in a conventional wheat cream medium supplemented with yeast powder and kept in the laboratory at a temperature of 25±1.2 °C and a relative humidity of 70–80% on a 12:12 light/dark cycle. Surviving flies were transferred to fresh food vials every 2 days.

### Experimental design and treatment

The experiment was divided into seven groups: 1) high sugar diet group, 2) high sugar diet + 0.078 uM acarbose, 3) high sugar diet + 0.156 uM acarbose, 4) high sugar diet + 0.3125 uM acarbose, 5) high sugar diet + 1.25 uM acarbose, 6) high sugar diet + 2.5 uM acarbose, and 7) high sugar diet + 5 uM acarbose. In each group, the experiment was performed with a total of 100 flies, distributed among 5 vials (*N* = 20 per vial). In this study, the negative control group consisted of *Drosophila* treated with a 6% sugar diet. In this investigation, the concentration of acarbose was determined empirically based on our preliminary analyses. Initial assays utilizing concentrations of 20 uM, 10 uM, and 5 uM revealed inhibition activities in excess of 95%. Following dose reduction to concentrations of 2.5 uM and 1.25 uM, inhibition activities were 81.40 and 68.79%, respectively. As a result, a concentration of 5 uM was regarded optimal for the study's initial acarbose concentration. The details of the protocol are as follows:

1. Cut a cotton cloth into a circular shape that fits the bottom of the test vial (2.0 × 9.5 cm).
2. Place the cut cotton cloth on the bottom of the vial and press firmly with a pipette tip.
3. Pour 0.8 mL of the diet solution onto the cotton cloth.
4. Press firmly with a pipette tip to prevent the flies from getting stuck in the gaps.
5. Separate the flies into vials containing cotton soaked with water to prevent dehydration and starve them for two hours.
6. After 2 h of starvation, the flies were transferred to the test and fed for 4 h.
7. After the feeding period, the flies were transferred to an empty vial.
8. The flies were euthanized with 5% CO_2_ fumigation and then placed in the refrigerator at −20 °C for 10 min.

Preparation of the sample

1. After the flies were euthanized, they were transferred to a 1.5-mL microcentrifuge tube.
2. The fly samples were washed three times with 1.0 mL of 50 mM potassium phosphate buffer.
3. Fly samples were then homogenized with a pestle in 1.0 mL of 50 mM potassium phosphate buffer using.
4. Fly debris was removed from the homogenate by centrifugation at 10,000 rpm for 10 min at 4 °C.
5. The supernatant was transferred to new 1.5 mL microcentrifuge tubes and kept on ice during preparation for measurement of protein content and α-glucosidase activity.

### Measurement of protein content

1. The Bradford reagent was thoroughly mixed in its container and then brought to room temperature.
2. Protein standards were prepared in a buffer containing 0.125 to 1.0 mg/mL BSA.
3. 5 µL of the protein standards or samples were added to separate wells of the 96-well plate.
4. 250 µL of Bradford reagent was added to each well and mixed on a shaker for approximately 30 s.
5. Samples were incubated at room temperature for 10 min. The absorbance was then measured at 595 nm.
6. The absorbance of each standard was plotted against its protein concentration.
7. The protein concentration of the fly supernatant samples was determined by comparing the net A595 values to the standard curve.

### Measurement of α-Glucosidase activity

1. 50 µL of buffer was added to each well of the 96-well plate. 100 µL of buffer was added to the blank wells.
2. 50 µL of samples were added to each well of the 96-well plate, including the blank well.
3. 50 µL of 5 mM *p*-nitrophenyl-α-d-glucopyranoside was added to each well of the 96-well plate, excluding the blank well.
4. The samples were incubated at room temperature for 30 min. Then, the absorbance was measured at 405 nm.
5. The absorbance values of all samples were divided by the protein.
6. The inhibitory activity (I) was calculated according to the following formula:
  $$\text{Inhibition}\;\left(\%\right)=\left(\frac{\text{Abs}\;\text{of}\;\text{control}-\text{Abs}\;\text{of}\;\text{sample}}{\text{Abs}\;\text{of}\;\text{control}}\right)x\;100$$

Here, Abs of the control represents the value OD of the sample from the high-sugar diet group.

7. IC_50_ values were obtained from plots of percent inhibition versus log concentration of acarbose and calculated from the mean inhibition values using nonlinear regression analysis.

### Validation of the method

**Fig 1:**
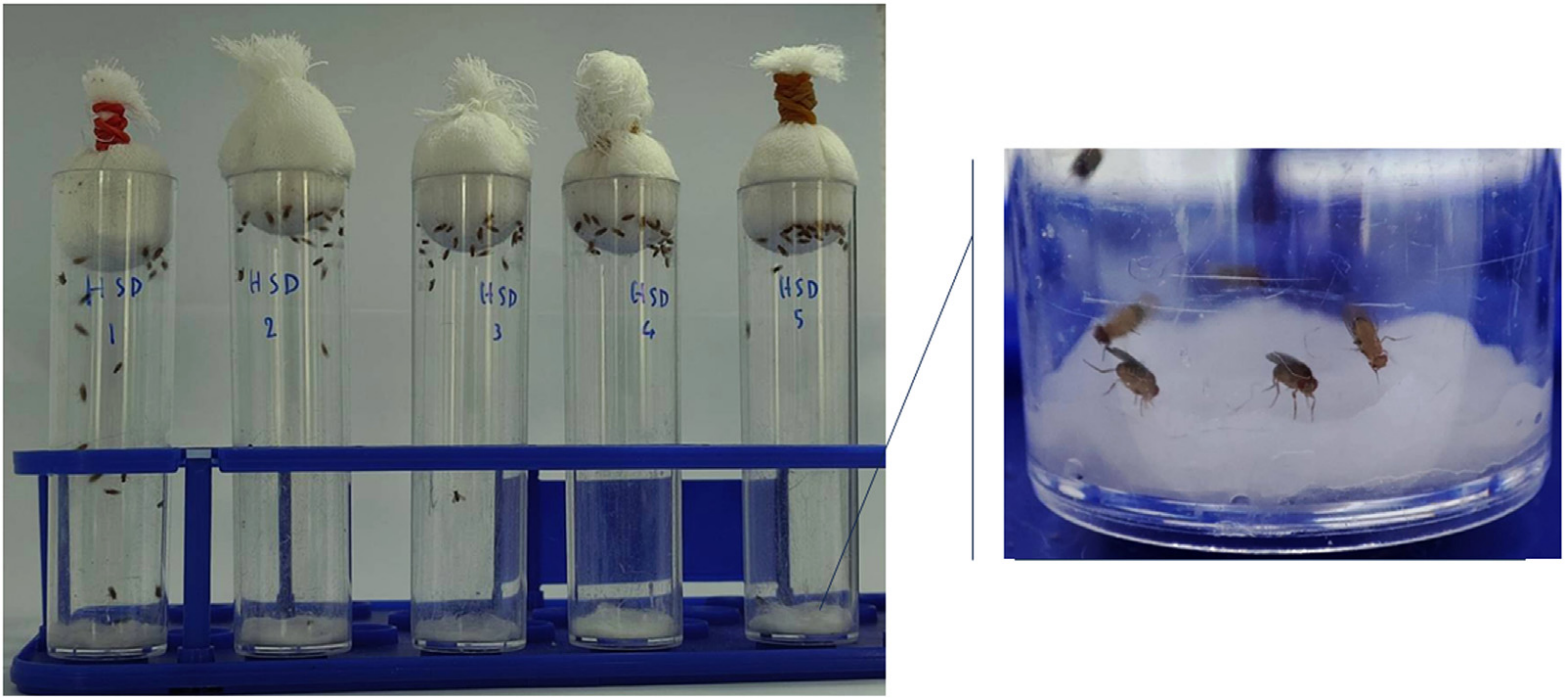
Feeding devices.

**Fig 2:**
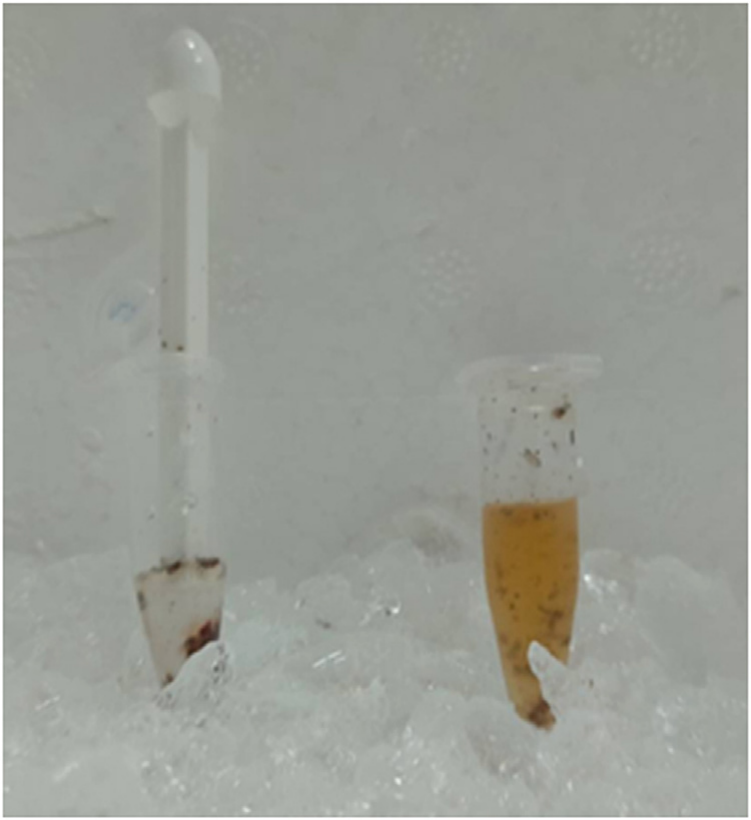
Sample preparation.

**Fig 3:**
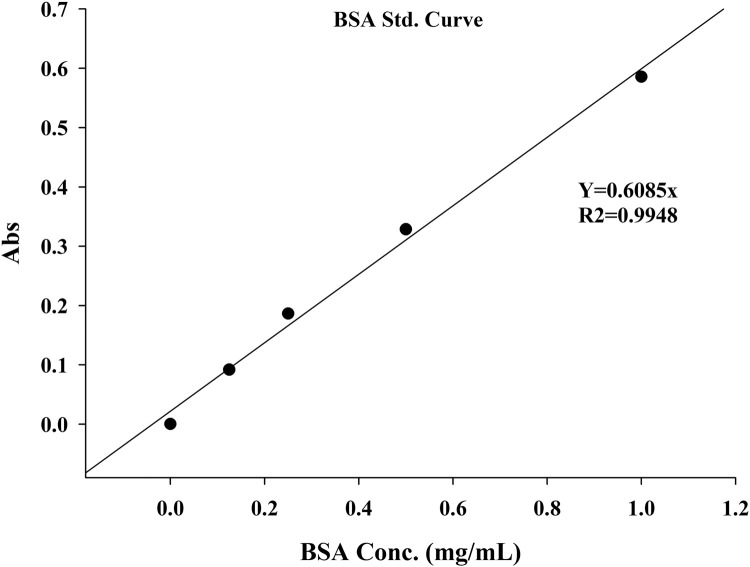
The standard curve of BSA.

**Fig 4:**
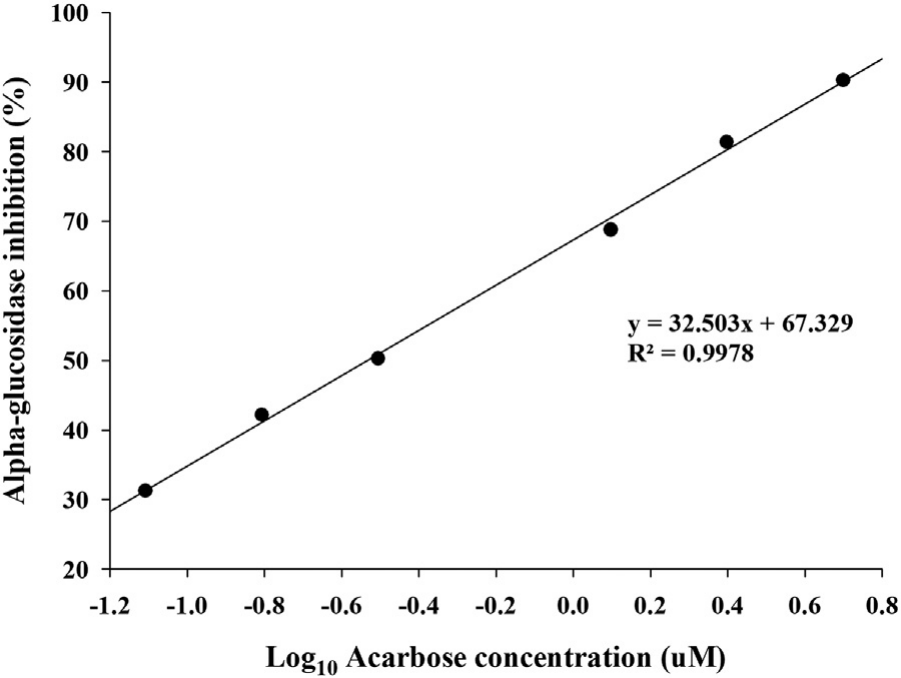
IC_50_ value of acarbose for inhibition of α-glucosidase.

In this study, we developed a rapid and inexpensive method to investigate the in vivo inhibition of α-glucosidase using wild-type female *D. melanogaster* of the Oregon-R-C strain at 8–10 days of age. To induce α-glucosidase activity, *Drosophila* were treated with a 30% sucrose solution. Acarbose served as a positive control to establish the method. Our results indicate that treatment with the 30% sucrose solution increased α-glucosidase activity by 15% compared with the control group treated with a 6% sucrose solution (*N* = 100, 20 per vial). The IC_50_ value for acarbose-mediated α-glucosidase inhibition in *Drosophila* was determined to be 0.587±0.014 µM (*N* = 100, 20 per vial). Thus, this method offers an efficient means of evaluating the potential of anti-diabetic compounds and provides finding that are more relevant to *in vivo* conditions than in vitro studies (Fig 1, Fig 2, Fig 3, Fig 4).

## Ethics statements

The animal research design was approved by the Ubon Ratchathani Rajabhat University's Ethic Committee (Ethical Clearance No. AN63008).

## Funding

This work was supported by the Research and Development Institute of Ubon Ratchathani Rajabhat University.

## CRediT authorship contribution statement

**Nattapong Wongchum:** Conceptualization, Methodology, Data curation, Writing – original draft. **Alongklod Tanomtong:** Conceptualization, Supervision, Writing – review & editing. **Somchai Pinlaor:** Supervision, Writing – review & editing. **Chatmongkon Suwannapoom:** Methodology. **Ananya Dechakhamphu:** Conceptualization, Methodology, Data curation, Writing – review & editing.

## Declaration of Competing Interest

The authors declare that they have no known competing financial interests or personal relationships that could have appeared to influence the work reported in this paper.

## Data Availability

Data will be made available on request.
